# Estimates of the severity of coronavirus disease 2019: a model-based analysis

**DOI:** 10.1016/S1473-3099(20)30243-7

**Published:** 2020-06

**Authors:** Robert Verity, Lucy C Okell, Ilaria Dorigatti, Peter Winskill, Charles Whittaker, Natsuko Imai, Gina Cuomo-Dannenburg, Hayley Thompson, Patrick G T Walker, Han Fu, Amy Dighe, Jamie T Griffin, Marc Baguelin, Sangeeta Bhatia, Adhiratha Boonyasiri, Anne Cori, Zulma Cucunubá, Rich FitzJohn, Katy Gaythorpe, Will Green, Arran Hamlet, Wes Hinsley, Daniel Laydon, Gemma Nedjati-Gilani, Steven Riley, Sabine van Elsland, Erik Volz, Haowei Wang, Yuanrong Wang, Xiaoyue Xi, Christl A Donnelly, Azra C Ghani, Neil M Ferguson

**Affiliations:** a MRC Centre for Global Infectious Disease Analysis, Abdul Latif Jameel Institute for Disease and Emergency Analytics, and Department of Infectious Disease Epidemiology, Imperial College London, London, UK; b School of Mathematical Sciences, Queen Mary University of London, London, UK; c Department of Statistics, University of Oxford, Oxford, UK

## Abstract

**Background:**

In the face of rapidly changing data, a range of case fatality ratio estimates for coronavirus disease 2019 (COVID-19) have been produced that differ substantially in magnitude. We aimed to provide robust estimates, accounting for censoring and ascertainment biases.

**Methods:**

We collected individual-case data for patients who died from COVID-19 in Hubei, mainland China (reported by national and provincial health commissions to Feb 8, 2020), and for cases outside of mainland China (from government or ministry of health websites and media reports for 37 countries, as well as Hong Kong and Macau, until Feb 25, 2020). These individual-case data were used to estimate the time between onset of symptoms and outcome (death or discharge from hospital). We next obtained age-stratified estimates of the case fatality ratio by relating the aggregate distribution of cases to the observed cumulative deaths in China, assuming a constant attack rate by age and adjusting for demography and age-based and location-based under-ascertainment. We also estimated the case fatality ratio from individual line-list data on 1334 cases identified outside of mainland China. Using data on the prevalence of PCR-confirmed cases in international residents repatriated from China, we obtained age-stratified estimates of the infection fatality ratio. Furthermore, data on age-stratified severity in a subset of 3665 cases from China were used to estimate the proportion of infected individuals who are likely to require hospitalisation.

**Findings:**

Using data on 24 deaths that occurred in mainland China and 165 recoveries outside of China, we estimated the mean duration from onset of symptoms to death to be 17·8 days (95% credible interval [CrI] 16·9–19·2) and to hospital discharge to be 24·7 days (22·9–28·1). In all laboratory confirmed and clinically diagnosed cases from mainland China (n=70 117), we estimated a crude case fatality ratio (adjusted for censoring) of 3·67% (95% CrI 3·56–3·80). However, after further adjusting for demography and under-ascertainment, we obtained a best estimate of the case fatality ratio in China of 1·38% (1·23–1·53), with substantially higher ratios in older age groups (0·32% [0·27–0·38] in those aged <60 years *vs* 6·4% [5·7–7·2] in those aged ≥60 years), up to 13·4% (11·2–15·9) in those aged 80 years or older. Estimates of case fatality ratio from international cases stratified by age were consistent with those from China (parametric estimate 1·4% [0·4–3·5] in those aged <60 years [n=360] and 4·5% [1·8–11·1] in those aged ≥60 years [n=151]). Our estimated overall infection fatality ratio for China was 0·66% (0·39–1·33), with an increasing profile with age. Similarly, estimates of the proportion of infected individuals likely to be hospitalised increased with age up to a maximum of 18·4% (11·0–37·6) in those aged 80 years or older.

**Interpretation:**

These early estimates give an indication of the fatality ratio across the spectrum of COVID-19 disease and show a strong age gradient in risk of death.

**Funding:**

UK Medical Research Council.

## Introduction

As of March 25, 2020, 414 179 cases and 18 440 deaths due to coronavirus disease 2019 (COVID-19), caused by the novel severe acute respiratory syndrome coronavirus 2 (SARS-CoV-2), had been reported worldwide.^1^ The epidemic began in mainland China, with a geographical focus in the city of Wuhan, Hubei. However, on Feb 26, 2020, the rate of increase in cases became greater in the rest of the world than inside China. Substantial outbreaks are occurring in Italy (69 176 cases), the USA (51 914 cases), and Iran (24 811 cases), and geographical expansion of the epidemic continues.

Clinical studies of hospitalised patients have shown that, at onset of COVID-19, patients frequently show symptoms associated with viral pneumonia, most commonly fever, cough, sore throat, myalgia, and fatigue.^2^, ^3^, ^4^, ^5^, ^6^ The case definition adopted in China and elsewhere includes further stratification of cases as severe (defined as tachypnoea [≥30 breaths per min], oxygen saturation ≤93% at rest, or PaO_2_/FiO_2_ ratio <300 mm Hg) and critical (respiratory failure requiring mechanical ventilation, septic shock, or other organ dysfunction or failure that requires intensive care).^7^ According to the report from the WHO–China Joint Mission on COVID-19, 80% of the 55 924 patients with laboratory-confirmed COVID-19 in China to Feb 20, 2020, had mild-to-moderate disease, including both non-pneumonia and pneumonia cases, while 13·8% developed severe disease and 6·1% developed to a critical stage requiring intensive care.^8^ In a study of clinical progression in 1099 patients,^4^ those at highest risk for severe disease and death included people over the age of 60 years and those with underlying conditions, including hypertension, diabetes, cardiovascular disease, chronic respiratory disease, and cancer.

**Research in context**

**Evidence before this study**

We searched PubMed, medRxiv, bioRxiv, arXiv, SSRN, Research Square, Virological, and Wellcome Open Research for peer-reviewed articles, preprints, and research reports on the severity of coronavirus disease 2019 (COVID-19), using the search terms “coronavirus”, “2019-nCoV”, and similar terms, and “fatality”, up to March 6, 2020. Several studies have estimated the case fatality ratio (the percentage of individuals with symptomatic or confirmed disease who die from the disease) and infection fatality ratio (the percentage of all infected individuals who die from the disease, including those with mild disease) of COVID-19 using a range of different statistical and modelling methods. Studies done solely in hospitalised patients report the highest fatality ratios (8–28%), representing the outcome for the most severely ill patients. Estimates of the population-level case fatality ratio from all case reports are in the range of 2–8%. Estimates of the infection fatality ratio averaged across all age-groups range from 0·2% to 1·6%, while estimates of the infection fatality ratio in the oldest age group (≥80 years) range from 8% to 36%. None of the identified studies had adjusted for differences in the denominator populations to obtain estimates that could be applied across populations. No other studies have estimated the proportion of infected individuals who will require hospitalisation.

**Added value of this study**

By synthesising data from across a range of surveillance settings, we obtained estimates of the age-stratified case fatality ratio and infection fatality ratio that take into account the different denominator populations in the datasets. Our underlying assumption, that attack rates (ie, the probability of becoming infected) do not vary substantially by age, is consistent with previous studies for respiratory infections. Under this assumption, differences in age patterns among cases in Wuhan versus those elsewhere in China would probably due to under-ascertainment of cases, given the different surveillance systems in place. Our results are consistent with this hypothesis, with cases in Wuhan seen in older individuals, who would have been identified through attendance at hospital, whereas cases elsewhere in China being younger overall, which would be explained by the policy of testing those with a travel history to Wuhan. After correcting for these biases, we found that estimates of the case fatality ratio from China are consistent with those obtained from early international cases. Our age-stratified estimates of the infection fatality ratio can be applied to any demography to give an estimate of the infection fatality ratio in older and younger populations. These estimates can be combined with estimates of the infection attack rate (approximately 80% for an unmitigated epidemic) to give rough projections of scale. Similarly, our estimates of the proportion of infections requiring hospitalisation can be combined with the infection attack rate to forecast health-care requirements.

**Implications of all the available evidence**

Our estimates of the case fatality ratio for COVID-19, although lower than some of the crude estimates made to date, are substantially higher than for recent influenza pandemics (eg, H1N1 influenza in 2009). With the rapid geographical spread observed to date, COVID-19 therefore represents a major global health threat in the coming weeks and months. Our estimate of the proportion of infected individuals requiring hospitalisation, when combined with likely infection attack rates (around 50–80%), show that even the most advanced health-care systems are likely to be overwhelmed. These estimates are therefore crucial to enable countries around the world to best prepare as the global pandemic continues to unfold.

Assessing the severity of COVID-19 is crucial to determine the appropriateness of mitigation strategies and to enable planning for health-care needs as epidemics unfold. However, crude case fatality ratios obtained by dividing the number of deaths by the number of cases can be misleading.^9^, ^10^ First, there can be a period of 2–3 weeks between a person developing symptoms, the case subsequently being detected and reported, and observation of the final clinical outcome. During a growing epidemic, the final clinical outcome of most of the reported cases is typically unknown. Simply dividing the cumulative reported number of deaths by the cumulative number of reported cases will therefore underestimate the true case fatality ratio early in an epidemic.^9^, ^10^, ^11^ This effect was observed in past epidemics of respiratory pathogens, including severe acute respiratory syndrome (SARS)^12^ and H1N1^9^ influenza, and as such is widely recognised. Thus, many of the estimates of the case fatality ratio that have been obtained to date for COVID-19 correct for this effect.^13^, ^14^, ^15^, ^16^ Additionally, however, during the exponential growth phase of an epidemic, the observed time lags between the onset of symptoms and outcome (recovery or death) are censored, and naive estimates of the observed times from symptom onset to outcome provide biased estimates of the actual distributions. Ignoring this effect tends to bias the estimated case fatality ratio downwards during the early growth phase of an epidemic.

**Figure 1:**
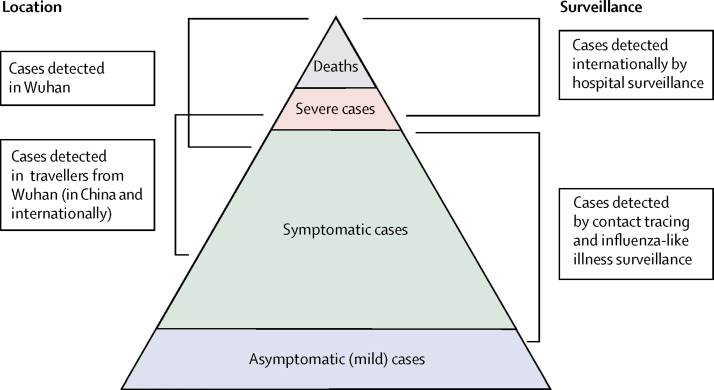
Spectrum of COVID-19 cases At the top of the pyramid, those meeting the WHO case criteria for severe or critical cases are likely to be identified in the hospital setting, presenting with atypical viral pneumonia. These cases will have been identified in mainland China and among those categorised internationally as local transmission. Many more cases are likely to be symptomatic (ie, with fever, cough, or myalgia), but might not require hospitalisation. These cases will have been identified through links to international travel to high-risk areas and through contact-tracing of contacts of confirmed cases. They might also be identified through population surveillance of, for example, influenza-like illness. The bottom part of the pyramid represents mild (and possibly asymptomatic) cases. These cases might be identified through contact tracing and subsequently via serological testing.

Second, surveillance of a newly emerged pathogen is typically biased towards detecting clinically severe cases, especially at the start of an epidemic when diagnostic capacity is low (figure 1). Estimates of the case fatality ratio can thus be biased upwards until the extent of clinically milder disease is determined.^9^ Data from the epicentre of the outbreak in Wuhan have primarily been obtained through hospital surveillance and, thus, are likely to represent patients with moderate or severe illness, with atypical pneumonia or acute respiratory distress being used to define suspected cases eligible for testing.^7^ In these individuals, clinical outcomes are likely to be more severe, so any estimates of the case fatality ratio will be higher. Elsewhere in mainland China and the rest of the world, countries and administrative regions alert to the risk of infection being imported via travel initially instituted surveillance for COVID-19 with a broader set of clinical criteria for defining a suspected case. These criteria typically included a combination of symptoms (eg, cough and fever) combined with recent travel history to the affected region (Wuhan, or Hubei province)^2^, ^17^. Such surveillance is likely to detect clinically mild cases but, by initially restricting testing to those with a travel history or link, might have missed other symptomatic cases.

Here we attempt to adjust for these biases in data sources to obtain estimates of the case fatality ratio (proportion of all cases that will eventually lead to death) and infection fatality ratio (the proportion of all infections that will eventually lead to death) using both individual-level case report data and aggregate case and death counts from mainland China, from Hong Kong and Macau, and international case reports. By adjusting for both underlying demography and potential under-ascertainment at different levels of the severity pyramid (figure 1), these estimates should be broadly applicable across a range of settings to inform health planning while more detailed case data accrue.

## Methods

### Individual-level data on early deaths from mainland China

We identified information on the characteristics of 48 patients who died from COVID-19 in Hubei, reported by the National Health Commission and the Hubei Province Health Commission website up to Feb 8, 2020. We recorded the following data elements, where available: sex, age, date of symptom onset, date of hospitalisation, and date of death. Of the 48 cases, neither the date of symptom onset nor the date of report was available for 13 cases. We also removed eight cases with onset before Jan 1, 2020, or death before Jan 21, 2020, and three deaths after Jan 28, 2020, which were the dates consistent with reliable reporting of onset and death in this setting, respectively, considering the onset-to-death times (including early onsets creates a bias towards long onset-to-death times, reflecting under-ascertainment of deaths early on). This left 24 deaths, which we used to estimate the onset-to-death distribution.

### Individual-level data on cases outside mainland China

We collated data on 2010 cases reported in 37 countries and two special administrative regions of China (Hong Kong and Macau), from government or ministry of health websites and media reports, until Feb 25, 2020. We recorded the following information where available: country or administrative region in which the case was detected, whether the infection was acquired in China or abroad, date of travel, date of symptom onset, date of hospitalisation, date of confirmation, date of recovery, and date of death. We used data from 165 recovered individuals with reported recovery dates and reported or imputed onset dates to estimate the onset-to-recovery distribution, after excluding 26 recoveries without appropriate information on dates of recovery, report, or locality. We used data on 1334 international cases to obtain estimates of the case fatality ratio, not including cases without dates of report.

### Data on aggregate cases and deaths in mainland China

Data on 70 117 PCR-confirmed and clinically diagnosed cases by date of onset in Wuhan and elsewhere in China from Jan 1 to Feb 11, 2020, were extracted from the WHO–China Joint Mission report.^8^ Over this period a total of 1023 deaths were reported across China, with these data available disaggregated into 10-year age bands between 0–9 years and 70–79 years old, and a further age band for those aged 80 years or older.^7^ Using collated data on daily reported deaths obtained each day from the National Health Commission regional websites, we estimated that 74% of deaths occurred in Wuhan and the remainder outside Wuhan. Additionally, the most recent available cumulative estimates (March 3, 2020) of 80 304 confirmed cases and 2946 deaths within China were extracted from the WHO COVID-19 Situation Report (number 43).^1^

An earlier (now withdrawn) preprint of a subset of these cases up to Jan 26, 2020 reported the age distribution of cases categorised by severity for 3665 cases.^18^ Under the China case definition, a severe case is defined as tachypnoea (≥30 breaths per min) or oxygen saturation 93% or higher at rest, or PaO_2_/FiO_2_ ratio less than 300 mm Hg.^7^ Assuming severe cases to require hospitalisation (as opposed to all of the patients who were hospitalised in China, some of whom will have been hospitalised to reduce onward transmission), we used the proportion of severe cases by age in these patients to estimate the proportion of cases and infections requiring hospitalisation.

### Data on infection in repatriated international Wuhan residents

Data on infection prevalence in repatriated expatriates returning to their home countries were obtained from government or ministry of health websites and media reports. To match to the incidence reported in Wuhan on Jan 30, 2020, we used data from six flights that departed between Jan 30 and Feb 1, 2020, inclusive.

### Data on cases and deaths on the *Diamond Princess* cruise ship

In early February 2020 a cruise liner named the *Diamond Princess* was quarantined after a disembarked passenger tested positive for the virus. Subsequently all 3711 passengers on board were tested over the next month. We extracted data on the ages of passengers onboard on Feb 5, 2020, the dates of positive test reports, which were available for 657 out of 712 PCR-confirmed cases, and the dates of ten deaths among these cases from the reports of the Japan Ministry of Health, Labour and Welfare^19^ and international media.

### Demographic data

Age-stratified population data for 2018 were obtained from the National Bureau of Statistics of China.^20^ According to these data, the population of Wuhan in 2018 was approximately 11 million people.

### Statistical analysis overview

All analyses were done with R software (version 3.6.2), with Bayesian Marko-Chain Monte Carlo via the package drjacoby (version 1.0.0).^21^ Data and code are available online at GitHub.

### Estimation of time intervals between symptom onset and outcome

In estimating time intervals between symptom onset and outcome, it was necessary to account for the fact that, during a growing epidemic, a higher proportion of the cases will have been infected recently (appendix p 7). Therefore, we re-parameterised a gamma model to account for exponential growth using a growth rate of 0·14 per day, obtained from the early case onset data (appendix p 6). Using Bayesian methods, we fitted gamma distributions to the data on time from onset to death and onset to recovery, conditional on having observed the final outcome. Missing onset dates were imputed on the basis of dates of report, where available.

### Estimation of case fatality ratio, infection fatality ratio, and proportion hospitalised from aggregated case data

Estimates of the distribution of times from onset-to-death were used to project the expected cumulative number of deaths given the onsets observed in Wuhan and outside Wuhan, assuming a uniform attack rate across age groups. Using the age-distribution of the population, we obtained an estimate of the expected number of infections in each age group. Under-ascertainment was estimated in and outside of Wuhan by comparing the number of observed cases by age to this expected distribution, assuming perfect ascertainment in the 50–59 age group as this group had the highest number of detected cases relative to population size. We also did a sensitivity analysis assuming a differential attack rate by age (appendix p 9). For Wuhan, we added scaling to account for further under-ascertainment compared with outside of Wuhan. These steps gave us the expected age-distribution of cases.

For a given onset-to-death distribution, we obtained a modelled estimate of the cumulative number of deaths by age under an age-dependent case fatality ratio (fitted relative to the case fatality ratio in the oldest age group, which represented the highest crude case fatality ratio). This estimate was compared with the observed deaths by age using a Poisson likelihood. These data were then jointly fitted alongside the most recent age-aggregated cumulative deaths and cases in mainland China. Given that the numbers of observed cases and deaths have dropped substantially following a peak in late January, the ratio of current cumulative cases to current number of deaths, once corrected for under-ascertainment, should provide a good estimate of the final case fatality ratio.^11^

To estimate the infection fatality ratio we fitted to data on infection prevalence from international Wuhan residents who were repatriated to their home countries. Our age-stratified case fatality ratio and infection fatality ratio model was jointly fitted to the case data and infection prevalence data with use of Bayesian methods, using our previous estimate of the onset-to-death distribution as a prior. Full mathematical details are provided in the appendix (p 8).

Assuming a uniform attack rate by age groups, we used the demography-adjusted under-ascertainment rates calculated above to obtain an estimate of the proportion of infected individuals who would require hospitalisation.

To independently validate our infection fatality ratio estimate, we analysed data from the outbreak on the *Diamond Princess* cruise liner taking the dates of reported positive tests as a proxy for onset date. We calculated the expected proportion of deaths observed until March 25, 2020, given the onset times and estimated onset-to-death distribution (appendix p 13).

### Estimation of case fatality ratio from individual case data

We used parametric and non-parametric methods^11^, ^22^ to estimate the case fatality ratio in cases reported outside of mainland China using individual-level data. Cases in which the outcome was unknown were treated as censored observations. For parametric and non-parametric analyses, missing onset dates were multiply imputed using information on the onset-to-report distribution, and unreported recoveries were imputed using onset-to-outcome distributions and country summary data. The parametric models were fitted to the data using Bayesian methods (appendix p 12).

### Role of the funding source

The funder of the study had no role in study design, data collection, data analysis, data interpretation, or writing of the report. The corresponding author had full access to all the data in the study and had final responsibility for the decision to submit for publication.

## Results

**Figure 2:**
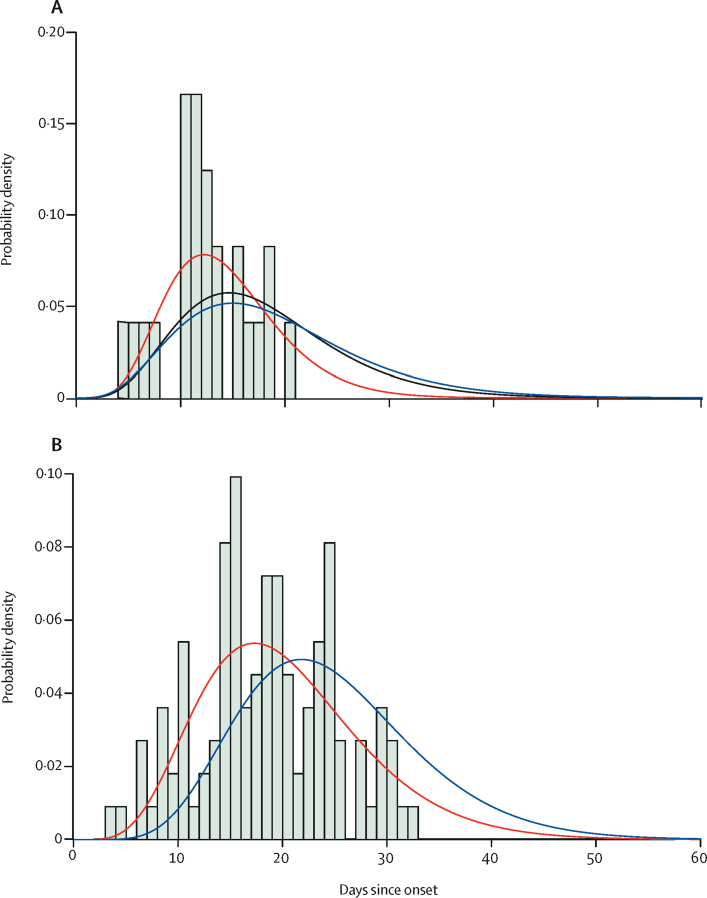
Onset-to-death and onset-to-recovery distributions (A) Onset-to-death data from 24 cases in mainland China early in the epidemic. (B) Onset-to-recovery data from 169 cases outside of mainland China. Red lines show the best fit (posterior mode) gamma distributions, uncorrected for epidemic growth, which are biased towards shorter durations. Blue lines show the same distributions corrected for epidemic growth. The black line (panel A) shows the posterior estimate of the onset-to-death distribution following fitting to the aggregate case data.

In the subset of 24 deaths from COVID-19 that occurred in mainland China early in the epidemic, with correction for bias introduced by the growth of the epidemic, we estimated the mean time from onset to death to be 18·8 days (95% credible interval [CrI] 15·7–49·7; figure 2) with a coefficient of variation of 0·45 (95% CrI 0·29–0·54). With the small number of observations in these data and given that they were from early in the epidemic, we could not rule out many deaths occurring with longer times from onset to death, hence the high upper limit of the credible interval. However, given that the epidemic in China has since declined, our posterior estimate of the mean time from onset to death, informed by the analysis of aggregated data from China, is more precise (mean 17·8 days [16·9–19·2]; figure 2).

Using data on the outcomes of 169 cases reported outside of mainland China, we estimated a mean onset-to-recovery time of 24·7 days (95% CrI 22·9–28·1) and coefficient of variation of 0·35 (0·31–0·39; figure 2). Both these onset-to-outcome estimates are consistent with a separate study in China.^23^

**Figure 3:**
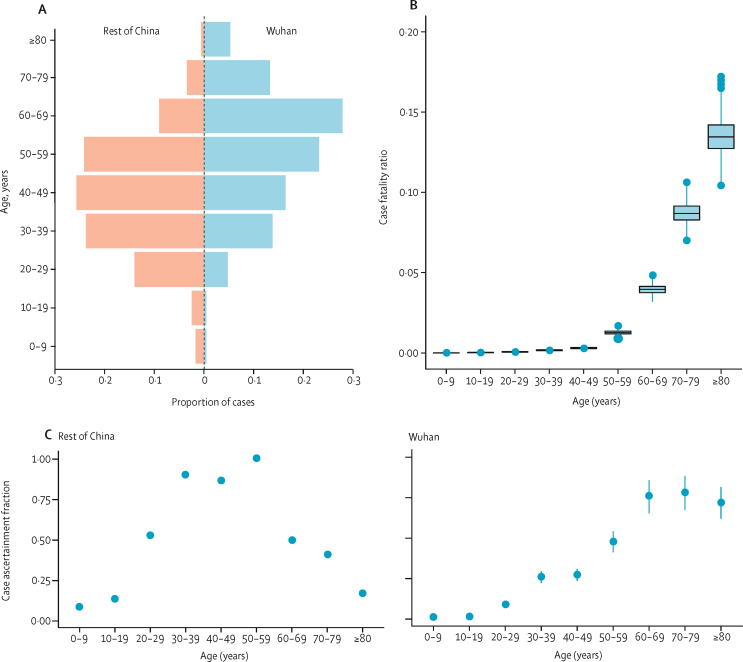
Estimates of case fatality ratio by age, obtained from aggregate data from mainland China (A) Age-distribution of cases in Wuhan and elsewhere in China. (B) Estimates of the case fatality ratio by age group, adjusted for demography and under-ascertainment. Boxes represent median (central horizontal line) and IQR, vertical lines represent 1·5 × IQR, and individual points represent any estimates outside of this range. (C) Estimated proportions of cases ascertained in the rest of China and in Wuhan relative to the 50–59 years age group elsewhere in China. Error bars represent 95% CrIs.

Case fatality ratios were estimated from aggregate data on cases and deaths in mainland China (table 1). A large proportion of the cases, including all of those early in the epidemic, were reported in Wuhan, where the local health system was quickly overwhelmed. As a result, the age distribution of cases reported in Wuhan differed to that in the rest of China (figure 3A). Reported cases in Wuhan were more frequent in older age groups, perhaps reflecting higher severity (and therefore prioritisation for hospitalisation in Wuhan), while cases outside of Wuhan might also show a bias in terms of the relationship between age and travel. Adjusting for differences in underlying demography and assuming no overall difference in the attack rate by age, we estimated high under-ascertainment of cases in younger age groups both inside and outside of Wuhan (figure 3C, D). Furthermore, we estimated a higher level of under-ascertainment overall in Wuhan compared with outside of Wuhan (figure 3C). Accounting for this under-ascertainment, we estimated the highest case fatality ratio (13·4% [11·2–15·9%]) in the 80 years and older age group (figure 3B, table 1), with lower case fatality ratios associated with lower age groups, and the lowest in the 0–9 years age group (0·00260% [0·000312–0·0382]).

**Table 1:** Estimates of case fatality ratio and infection fatality ratio obtained from aggregate time series of cases in mainland China

		**Deaths**	Laboratory-confirmed cases*	**Case fatality ratio**			**Infection fatality ratio**†
				Crude	Adjusted for censoring	Adjusted for censoring, demography, and under-ascertainment‡	
Overall		1023	44 672	2·29% (2·15–2·43)	3·67% (3·56–3·80)	1·38% (1·23–1·53)	0·657% (0·389–1·33)
Age group, years							
	0–9	0	416	0·000% (0·000–0·883)	0·0954% (0·0110–1·34)	0·00260% (0·000312–0·0382)	0·00161% (0·000185–0·0249)
	10–19	1	549	0·182% (0·00461–1·01)	0·352% (0·0663–1·74)	0·0148% (0·00288–0·0759)	0·00695% (0·00149–0·0502)
	20–29	7	3619	0·193% (0·0778–0·398)	0·296% (0·158–0·662)	0·0600% (0·0317–0·132)	0·0309% (0·0138–0·0923)
	30–39	18	7600	0·237% (0·140–0·374)	0·348% (0·241–0·577)	0·146% (0·103–0·255)	0·0844% (0·0408–0·185)
	40–49	38	8571	0·443% (0·314–0·608)	0·711% (0·521–0·966)	0·295% (0·221–0·422)	0·161% (0·0764–0·323)
	50–59	130	10 008	1·30% (1·09–1·54)	2·06% (1·74–2·43)	1·25% (1·03–1·55)	0·595% (0·344–1·28)
	60–69	309	8583	3·60% (3·22–4·02)	5·79% (5·20–6·34)	3·99% (3·41–4·55)	1·93% (1·11–3·89)
	70–79	312	3918	7·96% (7·13–8·86)	12·7% (11·5–13·9)	8·61% (7·48–9·99)	4·28% (2·45–8·44)
	≥80	208	1408	14·8% (13·0–16·7)	23·3% (20·3–26·7)	13·4% (11·2–15·9)	7·80% (3·80–13·3)
Age category (binary), years							
	<60	194	30 763	0·631% (0·545–0·726)	1·01% (0·900–1·17)	0·318% (0·274–0·378)	0·145% (0·0883–0·317)
	≥60	829	13 909	5·96% (5·57–6·37)	9·49% (9·11–9·95)	6·38% (5·70–7·17)	3·28% (1·82–6·18)

Crude case fatality ratios are presented as mean (95% confidence interval). All other fatality ratios are presented as posterior mode (95% credible interval). Estimates are shown to three significant figures. Cases and deaths are aggregate numbers reported from Jan 1 to Feb 11, 2020.^8^ Crude case fatality ratios are calculated as the number of deaths divided by the number of laboratory-confirmed cases. Our estimates also include clinically diagnosed cases (a scaling of 1·31 applied across all age-groups, as the breakdown by age was not reported for clinically diagnosed cases), which gives larger denominators and thus lower case fatality ratios than if only laboratory-confirmed cases were included.

* Values do not include the clinically diagnosed cases included in our estimates.

† Obtained by combining estimates of case fatality ratios with information on infection prevalence obtained from those returning home on repatriation flights.

‡ Accounts for the underlying demography in Wuhan and elsewhere in China and corrects for under-ascertainment.

In cases reported outside of mainland China, we estimated an overall modal case fatality ratio of 2·7% (95% CrI 1·4–4·7) using the parametric model (table 2). In those who reported travel to mainland China (and would therefore have been detected in the surveillance system), we estimated an overall modal case fatality ratio of 1·1% (0·4–4·1), and in those without any reported travel to China (therefore detected either through contact tracing or through hospital surveillance), we estimated a case fatality ratio of 3·6% (1·9–7·2) using the parametric model. The estimated case fatality ratio was lower in those aged under 60 years of age (1·4% [0·4–3·5]) compared with those aged 60 years and over (4·5% [1·8–11·1]). Similar estimates were obtained using non-parametric methods (table 2).

**Table 2:** Estimates of case fatality ratio obtained from individual-level data on cases identified outside of mainland China

		**Parametric**		**Non-parametric**	
		n	Case fatality ratio	n	Case fatality ratio
Overall		585	2·7% (1·4–4·7)	1334	4·1% (2·1–7·8)
Travel versus local transmission					
	Travellers to mainland China	203	1·1% (0·4–4·1)	208	2·4% (0·6–8·5)
	Local transmission	382	3·6% (1·9–7·2)	387	3·8% (1·7–8·2)
Age group, years					
	<60	360	1·4% (0·4–3·5)	449	1·5% (0·6–3·9)
	≥60	151	4·5% (1·8–11·1)	181	12·8% (4·1–33·5)

Parametric estimates are presented as posterior mode (95% credible interval), and were obtained using the gamma-distributed estimates of onset-to-death and onset-to-recovery. Non-parametric estimates are presented as maximum likelihood estimate (95% confidence interval) and were obtained using a modified Kaplan-Meier method.^11^, ^23^ Note that due to missing data on age and travel status, numbers in the stratified analysis are lower than for the overall analysis. In addition, the parametric method requires a correction for the epidemic growth rate, and these estimates were therefore obtained from the subset of data for which the travel or local transmission and age was known.

In international Wuhan residents repatriated on six flights, we estimated a prevalence of infection of 0·87% (95% CI 0·32–1·9; six of 689). Adjusting for demography and under-ascertainment, we estimate an infection fatality ratio of 0·66% (95% CrI 0·39–1·33). As for the case fatality ratio, this is strongly age-dependent, with estimates rising steeply from age 50 years upwards (table 1). The demography-adjusted and under-ascertainment-adjusted proportion of infected individuals requiring hospitalisation ranges from 1·1% in the 20–29 years age group up to 18·4% in those 80 years and older (table 3). Using these age-stratified infection fatality ratio estimates, we estimate the infection fatality ratio in the *Diamond Princess* population to be 2·9%. Given the delay from onset of symptoms to death, we would expect 97% of these deaths to have occurred by March 25, 2020, giving an estimate of the current infection fatality ratio of 2·8%, compared with the empirical estimate of 1·4% (95% CI 0·7–2·6; ten of 712).

**Table 3:** Estimates of the proportion of all infections that would lead to hospitalisation, obtained from a subset of cases reported in mainland China^18^

	**Severe cases**	**All cases**	**Proportion of infected individuals hospitalised**
0–9 years	0	13	0·00% (0·00–0·00)
10–19 years	1	50	0·0408% (0·0243–0·0832)
20–29 years	49	437	1·04% (0·622–2·13)
30–39 years	124	733	3·43% (2·04–7·00)
40–49 years	154	743	4·25% (2·53–8·68)
50–59 years	222	790	8·16% (4·86–16·7)
60–69 years	201	560	11·8% (7·01–24·0)
70–79 years	133	263	16·6% (9·87–33·8)
≥80 years	51	76	18·4% (11·0–37·6)

Proportions of infected individuals hospitalised are presented as posterior mode (95% credible interval) and are adjusted for under-ascertainment and corrected for demography. Estimates are shown to three signficant figures. We assumed, based on severity classification from a UK context, that cases defined as severe would be hospitalised.

## Discussion

From an extensive analysis of data from different regions of the world, our best estimate at the current time for the case fatality ratio of COVID-19 in China is 1·38% (95% CrI 1·23–1·53). Although this value remains lower than estimates for other coronaviruses, including SARS^24^ and Middle East respiratory syndrome (MERS),^25^ it is substantially higher than estimates from the 2009 H1N1 influenza pandemic.^26^, ^27^ Our estimate of an infection fatality ratio of 0·66% in China was informed by PCR testing of international Wuhan residents returning on repatriation flights. This value was consistent with the infection fatality ratio observed in passengers on the *Diamond Princess* cruise ship up to March 5, 2020, although it is slightly above the upper 95% confidence limit of the age-adjusted infection fatality ratio observed by March 25 (of 712 confirmed cases, 601 have been discharged, ten have died, and 11 remain in a critical condition). This difference might be due to repatriation flight data slightly underestimating milder infections, or due to cruise passengers having better outcomes because of a potentially higher-than-average quality of health care.

Our estimates of the probability of requiring hospitalisation assume that only severe cases require hospitalisation. This assumption is clearly different from the pattern of hospitalisation that occurred in China, where hospitalisation was also used to ensure case isolation. Mortality can also be expected to vary with the underlying health of specific populations, given that the risks associated with COVID-19 will be heavily influenced by the presence of underlying comorbidities.

Our estimate of the case fatality ratio is substantially lower than the crude case fatality ratio obtained from China based on the cases and deaths observed to date, which is currently 3·67%, as well as many of the estimates currently in the literature. The principle reason for this difference is that the crude estimate does not take into account the severity of cases. For example, various estimates have been made from patient populations ranging from those with generally milder symptoms (for example international travellers detected through screening of travel history)^13^ through to those identified in the hospital setting.^14^, ^15^

It is clear from the data that have emerged from China that case fatality ratio increases substantially with age. Our results suggest a very low fatality ratio in those under the age of 20 years. As there are very few cases in this age group, it remains unclear whether this reflects a low risk of death or a difference in susceptibility, although early results indicate young people are not at lower risk of infection than adults.^28^ Serological testing in this age group will be crucial in the coming weeks to understand the significance of this age group in driving population transmission. The estimated increase in severity with age is clearly reflected in case reports, in which the mean age tends to be in the range of 50–60 years. Different surveillance systems will pick up a different age case mix, and we find that those with milder symptoms detected through a history of travel are younger on average than those detected through hospital surveillance. Our correction for this surveillance bias therefore allows us to obtain estimates that can be applied to different case mixes and demographic population structures. However, it should be noted that this correction is applicable under the assumption of a uniform infection attack rate (ie, exposure) across the population. We also assumed perfect case ascertainment outside of Wuhan in the age group with the most cases relative to their population size (50–59-year-olds); however, if many cases were missed, the case fatality ratio and infection fatality ratio estimates might be lower. In the absence of random population surveys of infection prevalence, our adjustment from case fatality ratio to infection fatality ratio relied on repatriation flight data, which was not age specific. The reported proportion of infected individuals who were asymptomatic on the *Diamond Princess* did not vary considerably by age, supporting this approach, but future larger representative population prevalence surveys and seroprevalence surveys will inform such estimates further.

Much of the data informing global estimates of the case fatality ratio at present are from the early outbreak in Wuhan. Given that the health system in this city was quickly overwhelmed, our estimates suggest that there is substantial under-ascertainment of cases in the younger age groups (who we estimate to have milder disease) by comparison with elsewhere in mainland China. This under-ascertainment is the main factor driving the difference between our estimate of the crude case fatality ratio from China (3·67%) and our best estimate of the overall case fatality ratio (1·38%). The case fatality ratio is likely to be strongly influenced by the availability of health-care facilities. However surprisingly, although health-care availability in Wuhan was stretched, our estimates from international cases are of a similar magnitude, suggesting relatively little difference in health outcome. Finally, as clinical knowledge of this new disease accrues, it is possible that outcomes will improve. It will therefore be important to revise these estimates as epidemics unfold.

The world is currently experiencing the early stages of a global pandemic. Although China has succeeded in containing the disease spread for 2 months, such containment is unlikely to be achievable in most countries. Thus, much of the world will experience very large community epidemics of COVID-19 over the coming weeks and months. Our estimates of the underlying infection fatality ratio of this virus will inform assessments of health effects likely to be experienced in different countries, and thus decisions around appropriate mitigation policies to be adopted.

**This online publication has been corrected. The first corrected version first appeared at thelancet.com/infection on April 15, 2020 and the second on May 4, 2020**

## Data sharing

All data and code used in this study are available in a GitHub repository.
