# Correction to *Lancet Infect Dis* 2020; published online March 30. https://doi.org/10.1016/S1473-3099(20)30243-7

**DOI:** 10.1016/S1473-3099(20)30368-6

**Published:** 2020-06

*Verity R, Okell LC, Dorigatti I, et al. Estimates of the severity of coronavirus disease 2019: a model-based analysis.* Lancet Infect Dis *2020; published online March 30. https://doi.org/10.1016/S1473-3099(20)30243-7*—In this Article, reference 18 and the first sentence of the fourth paragraph in the Methods section have been updated to indicate that the cited preprint article was withdrawn. The authors believe that the data obtained from this referenced article (table 3) remain correct, as they are consistent with other published data. The reference citation for table 3 has also been corrected. These corrections have been made to the online version as of May 4, 2020, and will be made to the printed version.

