# Supplementary material for: Estimates of the severity of coronavirus disease 2019: a model-based analysis
Source: Lancet Infect Dis. 2020 Jun;20(6):669–77. doi: 10.1016/S1473-3099(20)30243-7 (PMC7158570; doi:10.1016/S1473-3099(20)30243-7)
Supplement: Supplementary appendix [file mmc1.pdf]

THE LANCET
Infectious Diseases

Supplementary appendix

This appendix formed part of the original submission and has been peer reviewed. We post it as supplied by the authors.

Supplement to: Verity R, Okell LC, Dorigatti I, et al. Estimates of the severity of coronavirus disease 2019: a model-based analysis. *Lancet Infect Dis* 2020; published onlineMarch 30. https://doi.org/10.1016/S1473-3099(20)30243-7.

Estimates of the severity of COVID-19 disease

Robert Verity[+1], Lucy C Okell[+1], Ilaria Dorigatti[+], Pete Winskill[+1], Charlie Whittaker[+1], Natsuko Imai[1], Gina Cuomo-Dannenburg[1], Hayley Thompson[1], Patrick GT Walker[1], Han Fu[1], Amy Dighe[1], Jamie T Griffin[2], Anne Cori[1], Marc Baguelin[1], Sangeeta Bhatia[1], Adhiratha Boonyasiri[1], Zulma Cucunubá[1], Rich FitzJohn[1], Katy Gaythorpe[1], Will Green[1], Arran Hamlet[1], Wes Hinsley[1], Daniel Laydon[1], Gemma Nedjati-Gilani[1], Steven Riley[1], Sabine van Elsland[1], Erik Volz[1], Haowei Wang[1], Yuanrong Wang[1], Xiaoyue Xi[1], Christl A Donnelly[1,3], Azra C Ghani[1*], Neil M Ferguson[1]*.

1. MRC Centre for Global Infectious Disease Analysis, J-IDEA; Department of Infectious Disease Epidemiology, Imperial College London
2. School of Mathematical Sciences, Queen Mary University of London
3. Department of Statistics, University of Oxford

Supplementary Material

1 Data Sources

1.1 Temporal Incidence Data for Wuhan and Rest of China

Epidemic curves from Figure 2 of the recent Report of the WHO-China Joint Mission on Coronavirus Disease 2019 (COVID-19)[8] were digitised and relevant data extracted using the openly available software DataThiefTM: incidence by symptom onset for the period spanning 8th December until 11th February were collated separately for Wuhan and the rest of China. Information on the age-distribution of cases and deaths over the same time period was also extracted from the recent China CDC Weekly Paper[7] – whilst the deaths were not stratified by location (Wuhan/rest of China), information scraped by volunteers at Imperial College from Chinese provincial Health Commission Reports enabled estimation of the proportion of deaths in China over that time period that had occurred in Wuhan. The observed cases across both locations were then scaled using a number of different adjustments to account for potential underreporting (detailed below). Throughout, we assume all deaths are completely ascertained (i.e. no missed deaths) after the 21st January, and that no detected deaths occurred before that date.

1.2 Individual-Level Data on International Cases

We collated individual line-list data from reports of international cases (see main text). The cases by country are summarised below.

International cases detected outside mainland China

Country/Administrative Region	Number of confirmed cases	Number of deaths	Number reported to have recovered
Afghanistan	1	-	0
Australia	22	-	15
Austria	2	-	0
Bahrain	17	-	0
Belgium	1	-	1
Cambodia	1	-	1
Canada	10	-	4
Croatia	1	-	0
Egypt	1	-	1
Finland	1	-	1
France	12	1	11
Germany	16	-	12
HK SAR	84	2	12

Italy	287	11	1
India	3	-	3
Iran	95	15	0
Iraq	5	-	0
Israel	2	-	0
Japan	163	1	23
Kuwait	8	-	0
Lebanon	1	-	0
Macau SAR	10	-	7
Malaysia	22	-	15
Nepal	1	-	1
Oman	4	-	0
Philippines	3	1	2
Russia	2	-	2
Singapore	91	-	58
South Korea	977	11	19
Spain	5	-	2
Sri Lanka	1	-	1
Sweden	1	-	0
Switzerland	1	-	0
Taiwan	31	1	2
Thailand	37	-	22
UAE	13	-	3
UK	9	-	8
USA	53	-	5
Vietnam	16	-	16

1.3 Prevalence Data from Repatriation Flights

Date on repatriation flights from Wuhan were collated from a number of different sources, including official Ministry of Health reports and media reports. From this data, we considered repatriation flights spanning a three-day period 30th January to 1st February (inclusive) - across these 3 days, a total of 689 individuals were repatriated from Wuhan on flights that tested all individuals (regardless of symptoms) for infection immediately upon arrivals. Testing following this repatriation yielded 6

positive individuals, a point prevalence of 0.87% - this estimate of point prevalence is then incorporated into the analyses detailed below to help estimate the extent of infection underreporting.

Destination	Date	Number Tested	Number Positive
Japan	30/01/2020	210	2
Japan	31/01/2020	149	2
Denmark	31/01/2020	4	0
France	31/01/2020	180	0
Germany	01/02/2020	115	2
Mongolia	01/02/2020	31	0
**Total**		**689**	**6**

1.4 Data from Diamond Princess Cruise Ship

We extracted data on the ages of passengers onboard on 5th February, the dates of reporting positive tests for 657 PCR-confirmed cases, and date of 7 deaths. These are shown below. NB some passengers were tested more than once, with a total of 4003 tests for 3711 passengers.

Date of report	N tested	N positive	References
05/02/2020	31	10	https://www.mhlw.go.jp/stf/newpage_09276.html
07/02/2020	171	41	https://www.mhlw.go.jp/stf/newpage_09340.html
08/02/2020	6	3	https://www.mhlw.go.jp/stf/newpage_09398.html
09/02/2020	57	6	https://www.mhlw.go.jp/stf/newpage_09405.html
10/02/2020	103	65	https://www.mhlw.go.jp/stf/newpage_09419.html
13/02/2020	221	44	https://www.mhlw.go.jp/stf/newpage_09425.html
13/02/2020	217	67	https://www.mhlw.go.jp/stf/newpage_09542.html
16/02/2020	289	70	https://www.mhlw.go.jp/stf/newpage_09547.html
17/02/2020	504	99	https://www.mhlw.go.jp/stf/newpage_09568.html
18/02/2020	681	88	https://www.mhlw.go.jp/stf/newpage_09606.html
19/02/2020	607	79	https://www.mhlw.go.jp/stf/newpage_09640.html
20/02/2020	52	13	https://www.mhlw.go.jp/stf/newpage_09668.html
23/02/2020	831	57	https://www.mhlw.go.jp/stf/newpage_09708.html
26/02/2020	167	14	https://www.mhlw.go.jp/stf/newpage_09783.html
02/03/2020	3	1	https://www.mhlw.go.jp/stf/newpage_09881.html

02/03/2020	63	0	https://www.mhlw.go.jp/stf/newpage_09881.html
**TOTALS**	**4003**	**657**	

We additionally use the age-distribution of the cases to estimate the IFR. These were available for 619 of the 712 cases; we assumed the age distribution in the remaining cases was the same. These are shown in the table below.

Age group (years)	Number of passengers	Number testing positive
0-9	16	1
10-19	23	5
20-29	347	28
30-39	429	34
40-49	333	27
50-59	398	59
60-69	924	177
70-79	1015	234
80-89	215	52
90-99	11	2
**Total**	**3711**	**619**

2 Statistical Methods

2.1 Intervals between onset of symptoms and death

Let t_o and t_d be the time (in days) of onset of symptoms and death, respectively, and let $\delta_{od} = t_d - t_o$ be the onset-to-death interval. If $f_{od}(\cdot)$ denotes the probability density function (PDF) of time from symptom onset to death, then the probability that a death on day t_d had onset of symptoms on day t_o is

$$g_{od}(t_o | \, t_d) = \frac{\int_{\delta_{od}}^{\delta_{od}+1} f_{od}(\tau) o(t_d - \tau) \, d\tau}{\int_0^{\infty} f_{od}(\tau') o(t_d - \tau') d\tau'} \, ,$$

where $o(t)$ denotes the observed number of onsets that occurred at time t. For an exponentially growing epidemic, we assume that $o(t) = o_0 e^{rt}$ where o_0 is the initial number of onsets (at $t = 0$) and *r* is the epidemic growth rate. Substituting this, we obtain

$$g_{od}(t_o|\, t_d) = \frac{\int_{\delta_{od}}^{\delta_{od}+1} f_{od}(\tau) e^{-r\tau}\, d\tau}{\int_0^{\infty} f_{od}(\tau') e^{-r\tau'} d\tau'} \ .$$

We can use this formula to fit the distribution $g_{od}(\cdot)$ to the observed data, correcting for the epidemic growth at rate r to estimate parameters of the true onset-to-death distribution $f_{od}(\cdot)$.

If we additionally assume that onsets were poorly observed prior to time $T_{\min}$ then we can include censoring:

$$g_{od}(t_o|\, t_d) = \frac{\int_{\delta_{od}}^{\delta_{od}+1} f_{od}(\tau) e^{-r\tau}\, d\tau}{\int_0^{t_d - T_{\min}} f_{od}(\tau') e^{-r\tau'} d\tau'} \ .$$

For the special case that we model $f_{od}(\cdot)$ as a gamma distribution parameterised in terms of its mean m_{od} and coefficient of variation s_{od} (defined as the ratio of the standard deviation to the mean), namely $f_{od}(\cdot\, |m_{od}, s_{od})$, it can be shown that

$$g_{od}\big(t_o|\, t_d, \hat{m_{od}}, \hat{s_{od}}\big) = \frac{\int_{\delta_{od}}^{\delta_{od}+1} f_{od}\big(\tau \mid m/\big(1 + r m_{od} s_{od}^2\big),\ s\big)\, d\tau}{\int_0^{t_d - T_{\min}} f_{od}(\tau' |\, m/(1 + r m_{od} s_{od}^2)\, , s) d\tau'} \ ,$$

where the transformed mean and standard deviation-to-mean ratios are $\hat{m_{od}} = \frac{m_{od}}{(1 + r m_{od} s_{od}^2)}, \hat{s_{od}} = s_{od}$.

The Bayesian posterior distribution for m_{od} and s_{od} is proportional to the product of this likelihood over a dataset of observed intervals and times of death $\{t_o, t_d\}$:

$$\Pr(m_{od}, s_{od}|\, \{t_o, t_d\}) \propto \prod_i g_{od}\big(t_{o,i}|\, t_{d,i}, \hat{m_{od}}, \hat{s_{od}}\big) \Pr(m_{od}, s_{od})\, ,$$

Here $\Pr(m_{od}, s_{od})$ is the joint prior distribution over m_{od} and s_{od}. We assume a $\mathrm{Uniform}(10{,}100)$ prior on m_{od} and a $\mathrm{Uniform}(0.2{,}0.8)$ prior on s_{od}, along with a fixed growth rate of $r = 0.14$. This growth rate was obtained by fitting a linear model to log(Wuhan cases) ~ date, using the temporal incidence data from Wuhan and focusing on the exponential-growth phase from 01/Jan/2020 to 19/Jan/2020. We note that higher growth rates will tend to elongate the estimated onset-to-outcome interval due to the assumption of greater bias in the data. We obtained the full posterior distributions of m_{od} and s_{od} by computing the joint distribution over a grid in increments of 0.05 and 0.005 respectively. We truncated the distribution by setting the likelihood to zero for combinations of m_{od} and s_{od} that generated gamma distributions with 95th percentile >100 days.

2.2 Intervals between onset of symptoms and recovery

Similar to the onset-to-death analysis above, we inferred the onset-to-recovery distribution $f_{or}(\cdot\, |m_{or}, s_{or})$ by fitting to data on the interval $\delta_{or} = t_r - t_o$ between onset of symptoms (t_o) and discharge from hospital (t_r). As above, we assumed a gamma distribution for $f_{or}(\cdot)$ resulting in an analytical expression for the epidemic-adjusted distribution $g_{or}(\cdot)$:

$$g_{or}\left(t_o \mid t_r, \hat{m_{or}}, \hat{s_{or}}\right) = \frac{\int_{\delta_{or}}^{\delta_{or}+1} f_{or}(\tau \mid m/(1 + rm_{or}s_{or}^2),\ s)\, d\tau}{\int_0^{t_r - T_{\min}} f_{or}(\tau' \mid m/(1 + rm_{or}s_{or}^2)\ , s) d\tau'}\ ,$$

where $\hat{m_{or}} = \frac{m_{or}}{(1+rm_{or}s_{or}^2)}, \hat{s_{or}} = s_{or}$.

We assumed r=0.14 in locally-acquired cases, consistent with growth in Wuhan. We used a lower growth rate of $r = 0.05$ for cases in travellers who had been infected in China, where the increase in case numbers had slowed and onsets were earlier; this growth rate gave onset-to-recovery estimates consistent with those in locally-acquired cases.

An added complication to this analysis was that many samples had missing onset dates. For samples with missing onset dates we assumed that symptom onset occurred prior to report date, i.e. $t_o = t_p - \varepsilon$, where t_p was the date of report (present in all cases) and ε was a free parameter. This resulted in an additional set of parameters $\varepsilon_1, \dots, \varepsilon_n$, where n is the number of cases with missing onset data. Note that when onset data are present, $\delta_{op} = t_p - t_o$ represents observed data, but when onset data are not present this reduces to $\delta_{op} = \varepsilon$. Assuming a gamma distribution for the onset-to-report distribution $f_{op}\left(\cdot \mid m_{op}, s_{op}\right)$ we obtain

$$g_{op}\left(t_o \mid t_p, \hat{m_{op}}, \hat{s_{op}}\right) = \frac{\int_{\delta_{op}}^{\delta_{op}+1} f_{op}\left(\tau \mid m/\left(1 + rm_{op}s_{op}^2\right),\ s\right) d\tau}{\int_0^{t_p - T_{\min}} f_{op}\left(\tau' \mid m/\left(1 + rm_{op}s_{op}^2\right), s\right) d\tau'},$$

where $\hat{m_{op}} = \frac{m_{op}}{(1+rm_{op}s_{op}^2)}, \hat{s_{op}} = s_{op}$.

This likelihoods from the two parts of this analysis were combined and multiplied by the prior to obtain

$$\begin{aligned}
&\Pr\left(m_{or}, s_{or}, m_{op}, s_{op}, \varepsilon_1, \dots, \varepsilon_n \mid \{t_o, t_p, t_r\}\right) \\
&\quad \propto \prod_i g_{or}\left(t_{o,i} \mid t_{r,i}, \hat{m_{or}}, \hat{s_{or}}\right) g_{op}\left(t_{o,i} \mid t_{p,i}, \hat{m_{op}}, \hat{s_{op}}, \varepsilon_1, \dots, \varepsilon_n\right) \times \\
&\qquad \Pr\left(m_{or}, s_{or}, m_{op}, s_{op}, \varepsilon_1, \dots, \varepsilon_n\right).
\end{aligned}$$

We assumed $\mathrm{Uniform}(10{,}100)$ priors on m_{or} and m_{op}, and $\mathrm{Uniform}(0.2{,}0.8)$ priors on s_{or} and s_{op}. We also assumed $\mathrm{Uniform}(0{,}50)$ priors on all ε parameters, which were treated as nuisance parameters when summarising other parameters. Due to the high dimensionality of this problem, parameters were estimated via MCMC in the R package drjacoby v1.0[1]

2.3 Epidemic growth-rate adjustment

Figure S1 below illustrates the requirement for the adjustment for epidemic growth for these onset-to-outcome distributions.

Figure S1: Adjusting onset-to-outcome distributions for epidemic growth. We simulated a growing epidemic up to day 60 (number of cases on day 1 = 2, growth rate=0.14, doubling time 5 days). The simulated onset-to-outcome distribution if everyone had been followed up until their outcome was observed is shown by the black bars whilst the onset-to-outcome distribution observed at day 60, censoring those whose outcome is not yet observed is shown by the red bars. The uncorrected Gamma distribution fitted to the observed outcome times at day 60 is shown in red and the Gamma

distribution fitted to the observed outcome times at day 60, corrected for epidemic growth, is shown in blue. The latter recovers the true distribution whilst the uncorrected fit results in distribution that is biased towards shorter durations.

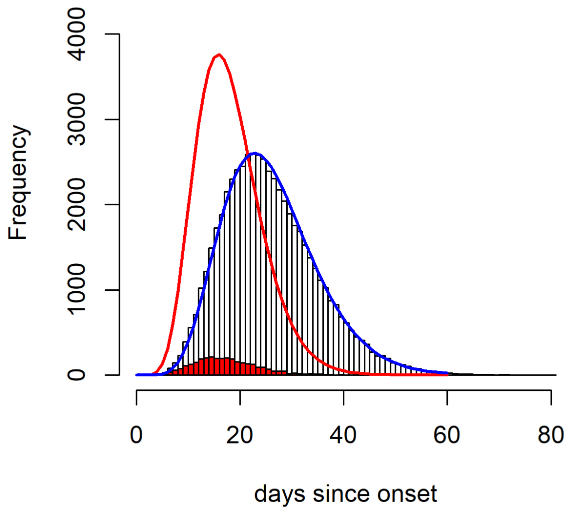

2.4 Age-stratified estimates of the Case Fatality Ratio and Infection Fatality Ratio from aggregate case data

2.4.1 Demographic adjustment

Assuming homogeneous attack rates across the different age groups, the demographic distribution of cases by age across each location should broadly match the demography of the populations in Wuhan and across the rest of China. The reported age-distribution of cases for both locations show striking deviations from the demographic structure of the Chinese populations. Wuhan, in particular, has noticeably fewer cases in younger age groups, and significant overrepresentation of older age-groups (see Figure 1B in main text). Similar patterns are evident in the age-distribution of cases outside China, but to a lesser extent. We hypothesised that these disparities were a product of under-ascertainment of cases, particularly in younger age-groups where a smaller proportion of infections would be expected to be severe and require hospitalisation.

In order to account for these disparities, we adjust the observed cases across both locations (inside Wuhan and outside Wuhan) to produce age-distributions of cases that matches China's demography. For each age-group and location, we calculate the following:

$$NC_{i,j} = \frac{Population_{i,j}}{Cases_{i,j}}$$

where i indexes each age-group and j indexes by location, and therefore $Population_{i,j}$ and $Cases_{i,j}$ describe the population and number of cases in age-group i and location j respectively. The reciprocal of $NC_{i,j}$ is therefore the attack-rate, which describes the number of cases per unit population.

This factor is then used to scale observed cases in the following way. For cases Outside Wuhan, we assume complete ascertainment in the age-group where the attack rate (highest valued reciprocal of

$NC_{i,j}$) is highest – that of the 50-59 year olds. We then adjust cases in the other age-groups to produce identical attack rates, so that for Outside Wuhan:

$$Adjusted\ Cases_{i,OutsideWuhan} = Cases_{i,OutsideWuhan} \max\left(\frac{1}{NC_{Outside\ Wuhan}}\right) NC_{i,Outside\ Wuhan}$$

We assume an additional level of under-ascertainment in Wuhan occurring due to the extensive strain on the health system, and so further scale the number of cases after the initial demographic adjustment above, such that

$$Adjusted\ Cases_{i,Wuhan} = Cases_{i,Wuhan} NC_{i,Wuhan} z$$

where z is a fitted parameter that is smaller than $\max\left(\frac{1}{NC_{i,j}}\right)$, implying that more cases are missed inside Wuhan than in the rest of the country.

We checked the sensitivity of our results to the assumption that there was under-ascertainment outside Wuhan. Under the alternative assumption that cases outside Wuhan are completely ascertained (and hence that the age-distribution observed reflects the true age distribution of cases outside of Wuhan) we obtained similar estimates (overall CFR 1.87% compared to 1.67% under our baseline assumption).

2.4.2 Statistical modelling framework

We worked within a Bayesian framework in order to jointly estimate the age-stratified case-fatality ratio, the onset-to-death distribution and the true underlying number of cases within Wuhan and other areas of mainland China, incorporating our prior knowledge of the onset-to-death distribution from fitting to observed data from 24 cases from mainland China (see Section2.1) .

Given our case and death age-stratification $A = \{a \in 1:9; 1 = 0 - 9\ years\ old, 2 = 10 - 19, \ldots 9 = 80 +\}$ we define the following parameters: the associated set of case-fatality rates θ_A, mean m_{od} and standard deviation to mean ratio s_{od} of the onset-to-death distribution $f_{od}(\cdot \mid m_{od}, s_{od})$. Observed cases are adjusted assuming homogeneous attack rates across age groups and a demographic age-distribution representative of China, assuming perfect case ascertainment in the 50-59 year old age group outside of Wuhan where there were the highest levels of case reporting relative to population size (see above). We also adjust for an additional level of underreporting specific to Wuhan (relative to elsewhere in China), z.

To fit these parameters we used the following data: D_W, the total observed deaths in Wuhan to 11th February 2020; D_A, the total observed deaths by age up to 11th February 2020, including those in Wuhan and $C_{T,A,L}$, observed cases by day, age and location up to this date. We also incorporated data on the total deaths and cases observed within mainland China by 4th March 2020 (without disaggregation by age or location), D_{M4} and C_{M4}.

$$\Pr\left(r_A, m_{od}, s_{od}, z, \varphi, r, D \middle| D_{M4}, C_{M4}, D_W, D_A, C_{T,A,L}, A_P, A_N\right) \propto L_1\ L_2\ L_3 \Pr(\theta_A) \Pr(m_{od}) \Pr(s_{od}) \Pr(z)$$

where

$$L_1 = \Pr\left(D_{M4} \middle| C_c, \theta_A, C'_{T,A,L}, m_{od}, s_{od}\right)$$

$$L_2 = \Pr\left(D_W, D_A | \theta_A, C'_{T,A,L}, m_{od}, s_{od}\right)$$

$$L_3 = \Pr\left(C'_{T,A,L}, I_{T,A,L} | C_{T,A,L}, A_P, \mathrm{A}_N, \omega, \varphi, r, D\right).$$

Here term L_1 represents the likelihood of the most recently observed crude case-fatality ratio (deaths/cases) in mainland China. The crude case fatality ratio tends to the true case fatality ratio as the proportion of the full epidemic which has been observed increases[2] and after adjustment for under ascertainment of cases. Cases in China have now reduced substantially relative to their late January 2020 peak. As such, this suggests that the recently estimated crude CFR likely represents a good approximation of the final epidemic CFR. Term L_2 represents the likelihood of the observed number of deaths in Wuhan (aggregated across age groups), and also, the observed number of deaths by age across all settings accounting for case-fatality rates by age, the epidemic curve adjusted for differences in ascertainment rates (by age and location) of cases and the distribution between case-onset and death. Term L_3 represents the model of how observed cases can be adjusted to reflect true cases, denoted $C'_{T,A,L}$, accounting for surveillance capacity in Wuhan, z, and age-based disparities in ascertainment throughout the course of the large-scale epidemic.

2.4.3 Estimation of infection rates from flight repatriation data

We also estimate infections, $I_{T,A,L}$ from true cases accounting for further under-ascertainment present across both locations. We inform this under-ascertainment of all infections using the observed prevalence of infections in travellers (n = 689) repatriated from Wuhan over the time period spanning 30th January – 1st February 2020 (inclusive). We estimate the prevalence of infection in Wuhan on 31st January by:

$$\text{Prevalence}_{\hat{T},W} = \frac{\frac{\varphi C'_{\hat{T},W}}{P_W}\left(1 - e^{-rD}\right)}{r}$$

where φ is an additional scaling factor for all infections, $C'_{\hat{T},W}$ is the estimated incidence of cases on 31st January in Wuhan (after the other age-based and Wuhan specific scaling detailed above), P_W is the population of Wuhan (assumed to be 11,081,000 people), r is the epidemic growth rate (assumed r = 0.14) and D is the detection window (duration that an infection remains detectable). For the age stratified analysis we assume Uniform priors on r of [0,0.1] and D of [7,14].

The remaining terms represent priors which were all uninformative with the exception of the onset-to-death parameters which were set to the likelihood surfaces estimated from the subset of observed onset to death durations.

2.4.4 Capturing age-stratified case-fatality ratios

Setting $T = 11^{\text{th}}$ of February 2020, the probability a case in age-category a with onset date t has died by time T is:

$$\lambda(a, t | \theta_a, m_{od}, s_{od}) = \theta_a \int_t^{T-t} f_{\mathrm{od}}(\tau \mid m_{od}, s_{od})\, d\tau$$

Assuming we observe $C'_{T,A,L}$, the true number of cases by day and age across all locations from the beginning of the epidemic $t_0 = 2^{\text{nd}}$ December (the date our data starts from), the expected number of deaths in age-category a is then:

$$E(D_a) = \sum_{t=t_0}^{T} \sum_{L} C'_{t,a,l}\, \lambda(a,t)\,.$$

We assume that observed deaths D_a follow a Poisson distribution with rate equal to the expectation $E(D_a)$:

$$\Pr\big(D_a \mid E(D_a)\big) = \frac{E(D_a)^{D_a} e^{-E(D_a)}}{D_a!}\,.$$

The likelihood of observing the full set of age-specific death-counts observed at T is then:

$$\Pr\big(D_A \mid \theta_A, C'_{T,A,L}, m_{od}, s_{od}\big) = \prod_{a\in A} \Pr\big(D_a \mid E(D_a)\big).$$

Simultaneously, the expected proportion of cases in Wuhan, π_w, can be assumed to follow a Binomial distribution (where $X{\sim}Bin(N,p)$ is the binomial distribution with X observations from N trials with probability p):

$$E(\pi_w) = \frac{\sum_{t=t_0}^{T} \sum_A C'_{t,a,w} \lambda(a,t)}{\sum_{t=t_0}^{T} \sum_A \sum_L C'_{t,a,l} \lambda(a,t)}\,,\;\; \Pr\big(D_w \mid \theta_A, C'_{T,A,L}, m_{od}, s_{od}\big) {\sim} Bin\big(\textstyle\sum_A D_a\,, E(\pi_w)\big)$$

As we assume the age-distribution and location of deaths are independent of one another:

$$\Pr\big(D_w, D_A \mid \theta_A, C'_{T,A,L}, m_{od}, s_{od}\big) = \Pr(D_w)\Pr(D_A).$$

2.4.5 Capturing post-peak overall case-fatality ratio

Given the total number of expected deaths across all-ages according to our age-stratified case-fatality ratios the overall number of expected deaths across all ages in China by T is:

$$E(D_{M4}) = \textstyle\sum_{a\in A} \sum_{t=t_0}^{T} \sum_L C'_{t,a}\, \lambda(a,t).$$

As cases in mainland China have been remained substantially lower than their late January 2020 peak since mid-February, current CFR estimates unadjusted by onset-to-death (i.e. true deaths to date divided by true cases to date) are likely to be a good estimator of the underlying CFR[2]. To capture this information, accounting for our estimates of the underlying surveillance capacity to capture all cases throughout the epidemic, we therefore assume that the current crude CFR in mainland China (i.e. current total deaths as a proportion of the current total observed cases) is a good estimate of the expected deaths arising from cases up to time T in China as a proportion of the unadjusted observed cases in this time period:

$$P\big(D_{M4} \big| C_{M4}, \theta_A, C'_{T,A,L}\big) {\sim} Bin\left(C_{M4}, \tfrac{E(D_T)}{C_T}\right),$$

where C_T is the total observed cases in China prior to time T (which is 11th February 2020).

2.5 Estimates of the Case Fatality Ratio from individual case data

2.5.1 Parametric estimates

Continuing our notation from section 2.1, let t_o, t_r and t_d denote the times of onset, recovery and death respectively, and let δ_{or} and δ_{od} denote onset-to-recovery and onset-to-death intervals. Additionally, let c denote the case-fatality ratio (CFR) such that each case has a probability c of ultimately resulting in death and a probability $(1-c)$ of ultimately resulting in recovery. For 72% of cases, the date of onset was not reported. For the cases with known date of reporting and missing onset date (n=958) we multiply imputed the dates of onset by jointly fitting a gamma distributed onset-to-report distribution. We also allow for imperfect identification of recoveries, such that each recovery has a probability p_r of being detected, and a probability $(1-p_r)$ of remaining in the data for an unlimited time as an un-coded or "other" event.

The probability that a patient dies on day t_d given onset at time t_o is given by:

$$\Pr(\text{outcome} = \text{death}, t_d |\, t_o, m_{od}, s_{od}, c) = c \int_{\delta_{od}}^{\delta_{od}+1} f_{od}(\tau \mid m_{od}, s_{od}) d\tau \,.$$

Similarly, the probability that a patient is detected as a recovery on day t_r, given onset at time t_o, or alternatively recovers but this recovery event is missed, is given by:

$$\Pr(\text{outcome} = \text{recovery}, t_r |\, t_o, m_{or}, s_{or}, c, p_r) = p_r(1-c) \int_{\delta_{or}}^{\delta_{or}+1} f_{or}(\tau \mid m_{or}, s_{or}) d\tau \,.$$

Finally, the probability that a patient remains in hospital, or that they recover but are not reported as a recovery, at the last date for which data are available, T, is

$$\Pr(\text{outcome} = \text{other} \mid t_o, m_{od}, s_{od}, m_{or}, s_{or}, c, p_r) = c \int_{T-t_o}^{\infty} f_{od}(\tau \mid m_{od}, s_{od}) d\tau +$$
$$p_r(1-c) \int_{T-t_o}^{\infty} f_{or}(\tau \mid m_{or}, s_{or}) d\tau +$$
$$(1-p_r)(1-c) \,.$$

The overall likelihood given all observed outcomes ($\text{outcome}_i \in \{\text{death}, \text{recovery}, \text{other}\}$) and corresponding outcome times (t_i) is simply the product over the individual terms:

$$\Pr\big(\{\text{outcome}_i, t_i\} \mid t_{o,i}, m_{od}, s_{od}, m_{or}, s_{or}, c, p_r\big)$$
$$= \prod_i \Pr(\text{outcome}_i, t_i \mid t_{o,i}, m_{od}, s_{od}, m_{or}, s_{or}, c, p_r) \,.$$

In a Bayesian context, the posterior distribution is obtained by multiplying this likelihood by the priors:

$$\Pr\big(m_{od}, s_{od}, m_{or}, s_{or}, c, p_r \mid \{\text{outcome}_i, t_{o,i}, t_i\}\big)$$
$$\propto \Pr\big(\{\text{outcome}_i, t_i\} \mid t_{o,i}, m_{od}, s_{od}, m_{or}, s_{or}, c, p_r\big) \times$$
$$\Pr(m_{od}, s_{od}) \Pr(m_{or}, s_{or}) \Pr(c) \Pr(p_r)$$

Here we have assumed that the joint prior can be decomposed into separate marginal priors on onset-to-death parameters, onset-to-recovery parameters, and separate priors on c and p_r. For the first two priors $\Pr(m_{od}, s_{od})$ and $\Pr(m_{or}, s_{or})$ we pass in the posterior distributions from the analyses above, namely the posterior m_{od} and s_{od} from the analysis in section 2.1, and the posterior m_{or} and s_{or} from the analysis in section 2.4.

2.5.2 Non-parametric estimates

To test sensitivity to parametric assumptions, we also estimated CFR in international cases using a modified Kaplan-Meier method (reference 25, main text) which accommodates the 3 possible outcomes (death, recovery, or censored), and does not assume any particular parametric distribution for onset-to-death and onset-to-recovery outcomes. Similarly to the parametric analysis, we multiply imputed missing onset times, now using observed onset-to-report times. Likewise, we imputed recovery status to allow for unreported recoveries. For this analysis, we used aggregated recovery numbers by country, which identified that 21% of total reported case recoveries at the country level were not recorded in the individual-level data because they could not be linked to a specific case. For each country, we used the number of unlinked recoveries to impute potential recoveries amongst those with no known outcome, sampling from these individuals weighted by the probability they had recovered given their onset date and the onset-to-recovery distribution from the parametric estimates.

2.6 Estimating the infection fatality ratio for the Diamond Princess data

We estimated the proportion of deaths amongst the passengers testing positive on the Diamond Princess that had occurred $\pi_{DP}(T)$ where T was the last date for which data are available (25th March 2020), given the probability density function (PDF) of time from symptom onset to death $f_{OD}(.)$:

$$E\big(\pi_{DP}(T)\big) = \sum_{i=1}^{N} \left(\int_{\tau=0}^{T-t_{0,i}} f_{OD}(\tau | \bar{m}_{od}, \bar{s}_{od}) d\tau \right) / N$$

where $\{t_{0,i}; 1..N\}$ are the set of time of onset in each of the N total number of test-positive individuals and $\bar{m}_{od}$ and $\bar{s}_{od}$ are the posterior mode of the mean and ratio of standard deviation to mean of onset duration distribution obtained from our fitting of these distributions to data from mainland China.

Figure S2 below shows the proportion first reported test-positive on each date according to Ministry of Health reports, and the fitted logistic growth curve.t We used the date of positive test report as a proxy for onset date, (acknowledging that this could be before the onset of symptoms for some passengers and after onset for others given potential delays in testing and reporting). Initially only symptomatic individuals were tested whilst later testing was extended to all passengers.

Figure S2 Proportion of tests positive by date on the Diamond Princess cruise ship. Data (points) and fitted logistic growth curve (log odds[proportion positive] = a + b*days, where a=-3.98, b=0.43 and days=days since the first positive test on 5th February up to tests on 2nd March) weighted by inverse variance of each data point.

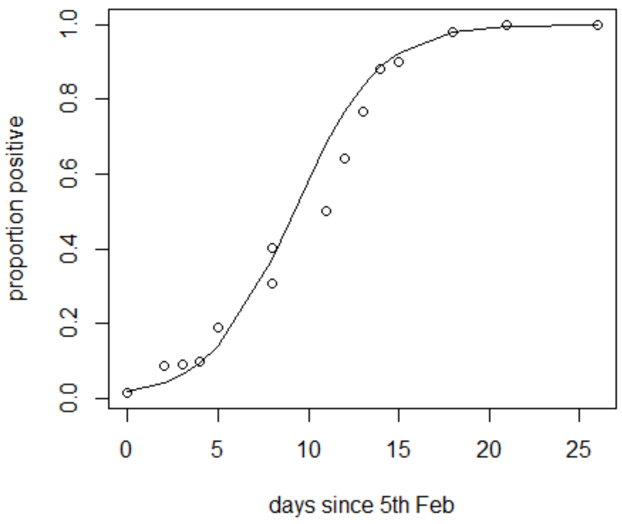

3 References

1 Verity R, Winskill P. drjacoby v1.0. 2020. https://mrc-ide.github.io/drjacoby/index.html.

2 Ghani AC, Donnelly CA, Cox DR, *et al.* Methods for estimating the case fatality ratio for a novel, emerging infectious disease. *Am J Epidemiol* 2005; **162**: 479–86.
